# Trends and geographical variation in mortality from coronary disease in Peru

**DOI:** 10.1371/journal.pone.0273949

**Published:** 2022-09-06

**Authors:** Diego Chambergo-Michilot, Noé Atamari-Anahui, Pedro Segura-Saldaña, Ana Brañez-Condorena, Carlos Alva-Diaz, Daniel Espinoza-Alva

**Affiliations:** 1 CHANGE Research Working Group, Facultad de Ciencias de la Salud, Carrera de Medicina Humana, Universidad Científica del Sur, Lima, Perú; 2 Department of Cardiology Research, Torres de Salud National Research Center, Lima, Peru; 3 Universidad San Ignacio de Loyola, Vicerrectorado de Investigación, Unidad de Investigación para la Generación y Síntesis de Evidencias en Salud, Lima, Perú; 4 Ingeniería Biomédica, Facultad de Ciencias y Filosofía, Universidad Peruana Cayetano Heredia, Lima, Perú; 5 ADIECS Asociación para el Desarrollo de la Investigación Estudiantil en Ciencias de la Salud, Universidad Nacional Mayor de San Marcos, Lima, Perú; 6 Universidad Señor de Sipán, Chiclayo, Perú; 7 Servicio de Neurología, Departamento de Medicina y Oficina de Apoyo a la Docencia e Investigación (OADI), Hospital Daniel Alcides Carrión, Callao, Perú; 8 Red de Eficacia Clínica y Sanitaria (REDECS), Lima, Perú; 9 Servicio de Cardiología Clínica, Instituto Nacional Cardiovascular-INCOR, EsSalud, Lima, Perú; Public Library of Science, UNITED STATES

## Abstract

**Background:**

Coronary disease (CD) is the main cause of mortality worldwide. Data about trends and geographical variation in CD mortality is available in some American countries. This information varies among countries since CD risk factors frequencies vary.

**Objective:**

To describe the trend and geographical variation of coronary disease (CD) mortality in Peru, 2005–2017.

**Methods:**

Analysis of secondary data of the Peruvian Ministry of Health’s registry of deaths. We analyzed CD mortality. We described the absolute and relative frequency of deaths and age-standardized mortality rate (ASMR) by natural regions, departments, age, sex, and year. We also described the change of ASMR between two periods (2005–2010 vs. 2011–2017).

**Results:**

There were 64,721 CD deaths between 2005 and 2017 (4.12% among all deaths). The absolute frequency of CD deaths was 5,665 and 6,565 in 2005 and 2017, respectively. CD mortality was more frequent in men and older adults. The ASMR varied among natural regions, being higher in the Coast (19.61 per 100,000 inhabitants). The change between the two periods revealed that almost all departments reduced their ASMRs, except for Callao, Lambayeque, and Madre de Dios.

**Conclusion:**

CD mortality has increased in Peru. Mortality was higher in men and older adults, and it varied among departments. More political efforts are needed to reduce these trends.

## Introduction

According to the Global Burden of Disease Study, coronary disease (CD) accounted for 10.6 million new cases in 2017, and was the second cause of longest years lived with disability among cardiovascular diseases [1].

It was observed that 9.4 million people died from CD in 2016, which meant an increase of 3.2% since 2000 [2]. Previous reports support that mortality trends are heterogeneous between countries. CD mortality in the United Kingdom, a high-income country, decreased by 72% between 1979 and 2013 [3], and it may be explained by better access to interventionism procedures [4]. In contrast, low and middle-income countries (LMICs) have presented a large increase since 2000 [5]. This have been reported in South America [6]. Nevertheless, we cannot extrapolate information from one LMIC to others because trends could vary due to heterogeneity in health systems and the burden of risk factors, such as hypertension and dyslipidemia [7, 8]. Consequently, CD mortality should be studied in each country. Also, diabetes mortality in Peru have been estimated, and authors reported a worrying increase up to 2014 [9], nevertheless, these results cannot estimate the rates of CD mortality.

The high frequency of cardiovascular risk factors in Peru and the need to update mortality trends justify the study. Indeed, a multicenter cohort evidenced a low prevalence of cardiovascular health metrics in Peruvian adults [10]. A nationally representative survey reported that the prevalence of hypertension, diabetes, and obesity was 9.5%, 3.6%, and 22.7%, respectively, in 2018 [11]. Additionally, The Pan American Health Organization (PAHO) reported that the standardized mortality rate for cardiovascular diseases was 111.4 and 65.4 for men and women between 30 to 69 years, respectively [12]. However, there are not published comprehensive results about CD mortality in Peru. Therefore, we aimed to describe the trend and geographical variation in departments and natural regions of Peru. The benefit of analyzing information by natural regions comes from the fact the natural regions present common demographic characteristics in Peru.

## Materials and methods

### Study design and setting

This study is a secondary analysis of the Ministry of Health’s (MINSA, Spanish initials) mortality registry in Peru between 2005 and 2017.

Peru is a South American LMIC that is composed of three natural regions: Coast, Mountains, and Jungle. Politically, it is composed of 25 departments. There are differences between each natural region that can increase some cardiovascular factors. For example, living in a semi-urban area increases the risk of hypertension while living at altitude decreases it [13]. Different dietary patterns in each natural region can explain a lower prevalence of diabetes and hypertension in rural areas (Mountains, Jungle) compared to urban areas (Coast) [14].

### Data sources

During February 2020, we requested the registry of CD deaths from MINSA webpage (https://bit.ly/31Evjr4). To request this information, we introduced CD ICD-10 codes: I20 (angina pectoris), I21 (acute myocardial infarction), I22 (subsequent myocardial infarction), I23 (certain current complications following acute myocardial infarction), I24 (other acute ischemic heart diseases) and I25 (chronic ischemic heart disease).

MINSA registries all deaths through several sources: medical records from healthcare centers, Public Ministry’s registry, and the National Registry of Identification and Civil Status. The latter allows relatives to register a death using a certificate signed by a physician.

When a person dies, physicians fill in a certificate that is compiled at the regional government. Then, it is sent to MINSA. This information is independent of the following assurances: Universal Health Assurance (UHA), Social Security, Armed Forces, and Police, and the Private sector.

We excluded 68 foreign people who died in Peru.

### Study variables

The outcome was annual age-standardized mortality rate (ASMRs), which was calculated using the World Health Organization (WHO) standard population distribution and the direct method [15, 16]. Additionally it was calculated absolute death frequency, relative frequency (CD deaths divided by total deaths) and CD crude mortality rate (absolute frequency divided by expected population [17] by 100,000 inhabitants) (S1 Table).

### Data analysis

Mortality was analyzed by year, sex, department, natural region, and age groups. The sex and age groups were distributed as 0–29 years, 30–59 years, and ≥60 years. This distribution was obtained from the MINSA database, so it was not possible to obtain individual data.

Three types of analysis determined declining change in time in each department. Firstly, we selected two periods of time (2005–2010 and 2011–2017), and calculated the positive or negative change (percentage, %). It was separated into two periods of time, because in 2009 the Health Insurance Plan (Plan de Aseguramiento en Salud) was approved, which made it possible to cover the costs of care for various diseases, including coronary diseases ICD 10: I20-I25, and from the year 2010 just its progressive implementation in all departments of Peru [18].

Secondly, we estimated the relative frequencies in each department by year, then we plotted it (*twoway* command), and analyzed the trends using the *nptrend* command, which is based on Cuzick’s method [19], and determines if there is a significant change (p<0.05). Thirdly, we followed an ecological approach through the mixed lineal models (*xtmixed* command) similar to a previous study [20]. The primary unit was department-year, and the dependent variable was relative frequency of CD deaths. Independent variables were 25 *dummy* ones (for each department). The model was adjusted for year, and it included an unstructured covariance and random intercept defined by department. The association estimator was β coefficient and its confidence intervals (95% CI). We used STATA v.14 (College Station, TX: StataCorp LLC) for analyses.

### Ethical considerations

Data were not analyzed at an individual level, thus, there was no risk of identifying individuals. The protocol of this study was approved by the Research Ethics Committee of Universidad Nacional Mayor de San Marcos (code: 0023).

## Results

### Descriptive analysis

Along study period, the ASMR was 21.51 per 100,000 inhabitants. ASMR was in men (32.62 per 100,000 inhabitants) and in women (19.83 per 100,000 inhabitans). ASMR by age was 0–29 years (0.42 per 100,000 inhabitants), 30–59 years (3.32 per 100,000 inhabitants), and ≥60 years (17.78 per inhabitants). ASMR was highest in Coast departments (19.61 per 100,000 inhabitants). Jungle was the region with lowest ASMR (9.42 per 100,000 inhabitants) (S1 Table). Departments with the highest ASMRs (≥20 per 100,000 inhabitants) were Tumbes, Piura, Lambayeque, La Libertad, Pasco, Ica, and Arequipa (S2 Table).

### Mortality change

Change between the two periods revealed that almost all departments reduced their ASMRs. Junin, Ucayali, and Ayacucho showed the greatest reduction (mean = -49%). Three departments increased their ASMRs: Callao (+21.53%), Lambayeque (+19.57%), and Madre de Dios (+15.99%) (Table 1).

**Table 1:** Age-standardized mortality change (%) between two periods in Peru (2005–2010 & 2011–2017).

Department	ASMR change ^a^		
	2005–2010	2011–2017	Change (%)
Coast			
Callao	17·46	21·22	21·53
Ica	26·42	22·19	-16·01
La Libertad	22·87	22·52	-1·53
Lambayeque	22·02	26·33	19·57
Lima	18·65	16·14	-13·46
Moquegua	10·24	9·12	-10·94
Piura	30·17	26·41	-12·46
Tacna	19·13	10·21	-46·63
Tumbes	31·77	16·94	-46·68
Mountains			
Ancash	17·04	10·89	-36·09
Apurimac	10·62	5·58	-47·46
Arequipa	23·67	20·76	-12·29
Ayacucho	9·84	4·99	-49·29
Cajamarca	17·50	9·77	-44·17
Cusco	13·91	6·62	-52·41
Huancavelica	8·46	6·61	-21·87
Huanuco	19·61	11·72	-40·23
Junin	14·37	7·23	-49·69
Pasco	12·94	8·68	-32·92
Puno	12·91	8·25	-36·10
Jungle			
Amazonas	13·59	9·63	-29·14
Loreto	5·98	5·60	-6·35
Madre de Dios	8·57	9·94	15·99
San Martin	11·04	8·85	-19·84
Ucayali	21·32	10·78	-49·44

^a^ ASMR: age-standardized mortality rate.

**Fig 1:**
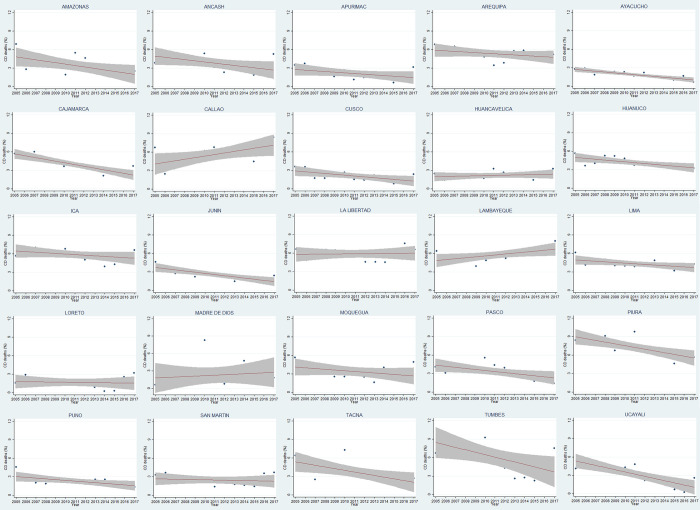
Mortality proportions (%) by coronary disease along years in each department in Peru.

Relative frequency of CD deaths significantly tended to low in Ayacucho, Cajamarca, Cusco, Huanuco, Junin, Pasco, Piura, Tacna, and Ucayali. We did not find statistical significance among departments whose frequencies appeared to increase (Fig 1).

There were several trends with significant positive and negative slopes. Positive: Arequipa, Callao, Huanuco, Ica, La Libertad, Lambayeque, Lima, Piura, and Tumbes. Negative: Apurimac, Ayacucho, Cusco, Huancavelica, Loreto, Madre de Dios, and Puno. The interpretation is as follows: the department of interest started the period (2005–2017) with a relative frequency of CD deaths β times higher or lower than the rest. The variable *year* showed that relative frequency decreased by 0.12 percentage points annually in Perú (Table 2).

**Table 2:** Mixed lineal models between department-year and proportion of deaths in departments in Peru (period 2005–2017).

Department	β	95% CI	P value
Amazonas	0·19	-0·64; 1·02	0·67
Ancash	0·6	-0·23; 1·43	0·158
Apurimac	-1·09	-1·92; -0·25	0·011
Arequipa	2·11	1·28; 2·95	<0·001
Ayacucho	-1·32	-2·15; -0·49	0·002
Cajamarca	0·74	-0·09; 1·57	0·081
Callao	2·39	1·56; 3·22	<0·001
Cusco	-1·13	-1·96; -0·29	0·008
Huancavelica	-1·03	-1·86; -0·20	0·015
Huanuco	0·94	0·10; 1·77	0·028
Ica	2·65	1·82; 3·48	<0·001
Junin	-0·63	-1·46; 0·20	0·138
La Libertad	2·69	1·85; 3·52	<0·001
Lambayeque	2·56	1·73; 3·39	<0·001
Lima	1·12	0·29; 1·95	0·008
Loreto	-1·53	-2·36; -0·67	<0·001
Madre de Dios	-0·93	-1·77; -0·10	0·028
Moquegua	0·19	-0·65; 1·02	0·661
Pasco	0·13	-0·70; 0·96	0·762
Piura	4·04	3·21; 4·87	<0·001
Puno	-0·92	-1·75; -0·09	0·03
San Martin	-0·7	-1·56; 0·10	0·084
Tacna	0·5	-0·33; 1·33	0·238
Tumbes	2·87	2·04; 3·70	<0·001

Ucayali was omitted due to collinearity.

## Discussion

### Main findings

For the period 2005–2017, we found heterogeneous ASMRs among departments, and the largest ones were located on the Coast. Relative frequency of CD deaths was 4.12%. Absolute frequency has increased by 15.89%. CD mortality is higher in men and older adults.

### Comparison with other studies

We observed that CD deaths increased by 15.89% in Peru. Similarly, the worldwide frequency has raised by 22.3% for the period 2007–2017 [21]. Incidence of cardiovascular risk factors, such as hypertension, smoking, and hypercholesterolemia, are determinants of CD dynamics in high-income countries and LMICs [22, 23]. The latter has been going through an epidemiological transition that challenges health systems [24].

Regarding Peru, burden of cardiovascular risk factors has grown. Diabetes mortality rates have increased from 11.7 to 17.2 per 100,000 inhabitants (2005–2014) [9]. Representative surveys reported that high blood pressure has slightly increased from 2014 to 2018 (9.5%-10.4%) [11]. Moreover, a multicenter cohort showed a high prevalence of poor cardiovascular metrics, such as unhealthy blood pressure, glucose, and lifestyles (31.8%) [10]. These results suggest that Peruvians are at risk of CD morbidity and mortality.

We observed that CD mortality was higher in men. Higher CD burden and mortality in men have been also reported in South America [6]. This may be attributable to sex disparities in risk factors. Studies in Peru and Latin countries have shown that multimorbidity, hypertension, smoking, and an unhealthy diet are more frequent in men [11, 25, 26]. Besides, medical controls of cardiovascular diseases are less common in men than in women [27]. Briefly, women’s estrogens have cardio-protective properties as it vasodilates systemic circulation, and enhance coronary endothelial function [28].

We observed that older adults had the highest mortality. Indeed, the WHO reported that CD mortality rises with aging, regardless of country income [29]. Cardiovascular diseases burden in elderly could have a role. For example, a Peruvian multicenter cohort found a low prevalence of ideal cardiovascular metrics in older adults [10].

We observed a decrease in CD mortality in the Mountains. We can partially explain it by comparing the trends of risk factors of mortality between natural regions. Atamari-Anahui et al, [9] reported that the lowest trends of diabetes mortality were located in the Mountains in comparison with Coast and Jungle. Experiences in other countries could support the important effect of changes in risk factors [30]. Peru is still on the way to health system decentralization, therefore MINSA deployed the UHA in 2009, which led to better preventive and recuperative services in all departments [31]. A posterior analysis showed that residing in the Mountains had a positive effect on healthcare access through UHA [32].

We observed that Jungle presented the lowest CD mortality. There are several factors that may explain it. First, previous studies stated an inverse association between altitude and some cardiovascular factors that influence CD mortality, such as hypertension [13] and obesity [33], however, altitude variation is too complex to assume this association. Compared with the other regions, the Jungle have less access to healthcare (less hospitals and primary care centers). This lack may reduce the capacity to detect cardiovascular risk factors, such as hypertension. Indeed, six out of 10 Jungle residents have undiagnosed hypertension [34]. Neglecting cardiovascular factors will create a false “healthy” region. Also, in the context of lack of healthcare access there could be an underestimation of CD-related mortality due to under-registration. The main consequence of under-reporting and a false “healthy” region is that health decision makers may neglect cardiovascular policies in that region due to a false “low” mortality. A key information that let us see that the Jungle region is not healthier than other ones is that it has a lower life expectancy than the Coast (Jungle: 69.9–72.9 vs. Coast: 71.1–79.9) [35].

### Relevance for public health

There is a tendency to decrease in different regions. Although, there is a need for better or new public health measures. Indeed, there is an action plan called “Plan of Action for the Prevention and Control of Noncommunicable Diseases in the Americas 2013–2019”, which settled the objective of reducing premature mortality of the main four non-communicable diseases and controlling cardiovascular risk factors, such as smoking, alcoholism, and hypertension [36]. However, we observed that CD mortality has increased in Peru since 2005, as well as risk factors burden [11].

There are international experiences that support action on risk factors reduction. Improvement in statins use, and outpatient monitoring partially explained the large decrease in CD mortality in Denmark (1991–2007) [37]. Indeed, other programs on hypertension, cholesterol, obesity, and diabetes care have focused on cultural and linguistic awareness to increase knowledge and prevent complications.

Future health policies based on dietary changes and education should be considered to diminish CD mortality. Peru has been working on some policies. In 2016, the Peruvian government approved the regulation that established the process of gradual reduction of trans-fats in food and non-alcoholic beverages. Two years later, the government approved the Advertising Warning Manual, which allows the identification of high-sodium products, sugar, and fats. These efforts are expected to modify CD mortality in the future years, being necessary to update our study.

## Limitations

Our study presents some limitations. Although the database is a national registry, it could present underreporting problems. For instance, while the decline of CD deaths from 2005 to 2006 was large (more than 1,000), the decline in the next years was quite lesser. It may reflect a problem in death registration out of public health establishments (i.e. private clinics). Mortality data from Social Security, Armed Forces and Police, and the Private sector could arrive late at MINSA, which may reduce the representativeness of its database on a determined date. Besides, multiple stages of data process introduce underreporting, so readers must consider this limitation. High quality of death forms filling was not ensured, so it should be considered as a limitation of the health personnel. We excluded 68 foreign people to ensure that our results have validity only for the Peruvian population. It was not possible to obtain the specific data to which region they belonged, nevertheless, they represented 0.1% of all deaths in the study period. Another limitation is that we did not analyze the influence of socioeconomic status on mortality, like Rosero-Bixby [38], did when analyzing mortality in elderly Costa Ricans. Socioeconomic status is a complex term that should not be determine by a whole department in Peru because each department has cities with heterogeneous economical activities that determine the socioeconomic status. We recommend that future papers address this variable (socioeconomic status) per a homogeneous location (not a whole department). Despite these limitations, our results provide relevant information that will support the implementation of more health policies.

In conclusion, we explored the Peruvian trends of CD mortality and analyzed the variations in natural regions, departments, age, and sex. CD deaths have increased since 2005. Mortality was higher in men and older adults.

## Supporting information

S1 Table Coronary disease deaths, crude mortality rate and age-standardized mortality by natural region, age group, sex, and year in Peru (period 2005–2017). (DOCX)

S2 Table CD deaths, crude mortality rate, and age-standardized mortality by departments in Peru (period 2005–2017). (DOCX)
