# Supplementary material for: Trends and geographical variation in mortality from coronary disease in Peru
Source: PLoS One. 2022 Sep 6;17(9):e0273949. doi: 10.1371/journal.pone.0273949 (PMC9447875; doi:10.1371/journal.pone.0273949)
Supplement: S1 Table — (DOCX) [file pone.0273949.s001.docx]

# S 1 Table. Coronary disease deaths, crude mortality rate and Age-standardized mortality by natural region, age group, sex, and year in Peru (period 2005-2017).

| **Variable** | **CD deaths** | **Proportion of CD deaths** | **Crude mortality rate** | **ASMR ^a^** |
| --- | --- | --- | --- | --- |
| Natural region |  |  |  |  |
| Coast | 44702 | 4·97 | 20·85 | 19·61 |
| Mountains | 17364 | 3·10 | 12·45 | 12·01 |
| Jungle | 2655 | 2·41 | 7·20 | 9·42 |
| Age group |  |  |  |  |
| 0-29 years | 1824 | 1·06 | 0·83 | 0·42 |
| 30-59 years | 11817 | 4·63 | 8·91 | 3·32 |
| ≥60 years | 51080 | 6·25 | 148·22 | 17·78 |
| Sex |  |  |  |  |
| Male | 37955 | 5·69 | 27·42 | 32·62 |
| Female | 26766 | 4·64 | 18·54 | 19·83 |
| Year |  |  |  |  |
| 2005 | 5665 | 5·49 | 20·37 | 30·09 |
| 2006 | 4605 | 4·38 | 16·36 | 23·52 |
| 2007 | 4708 | 4·39 | 16·53 | 23·37 |
| 2008 | 4903 | 4·54 | 17·02 | 23·65 |
| 2009 | 4496 | 4·06 | 15·43 | 21·02 |
| 2010 | 4575 | 4·23 | 15·53 | 20·77 |
| 2011 | 4758 | 4·02 | 15·97 | 20·79 |
| 2012 | 4410 | 3·69 | 14·63 | 18·70 |
| 2013 | 5207 | 4·15 | 17·09 | 17·21 |
| 2014 | 4959 | 3·73 | 16·09 | 19·81 |
| 2015 | 4181 | 3·10 | 13·42 | 16·20 |
| 2016 | 5689 | 3·91 | 18·07 | 20·07 |
| 2017 | 6565 | 4·38 | 20·63 | 23·82 |
| Overall | 64721 | 4·12 | 16·70 | 21·51 |

^a^ ASMR: age-standardized mortality rate. CD: Coronary disease.
