# Supplementary material for: Trends and geographical variation in mortality from coronary disease in Peru
Source: PLoS One. 2022 Sep 6;17(9):e0273949. doi: 10.1371/journal.pone.0273949 (PMC9447875; doi:10.1371/journal.pone.0273949)
Supplement: S2 Table — (DOCX) [file pone.0273949.s002.docx]

**S2 Table. CD deaths, crude mortality rate, and age-standardized mortality by departments in Peru (period 2005-2017).**

| **Department** | **Mean population by year** | **Proportion of CD deaths** | **CD deaths** | **Crude mortality rate** | **ASMR ^a^** |
| --- | --- | --- | --- | --- | --- |
| Coast |  |  |  |  |  |
| Callao | 948371 | 5·67 | 2523 | 20·46 | 19·67 |
| Ica | 752824 | 5·81 | 2561 | 26·17 | 23·77 |
| La Libertad | 1778293 | 5·90 | 5542 | 23·97 | 22·53 |
| Lambayeque | 1217187 | 5·85 | 3968 | 25·08 | 24·56 |
| Lima | 9272918 | 4·27 | 22497 | 18·66 | 17·04 |
| Moquegua | 175743 | 3·32 | 257 | 11·56 | 9·78 |
| Piura | 1793023 | 7·03 | 6267 | 25·22 | 26·14 |
| Tacna | 325885 | 3·47 | 540 | 12·96 | 13·71 |
| Tumbes | 224279 | 5·83 | 547 | 19·31 | 23·45 |
| Mountains |  |  |  |  |  |
| Ancash | 1136193 | 3·72 | 2171 | 14·70 | 13·40 |
| Apurimac | 464538 | 2·12 | 488 | 8·08 | 7·52 |
| Arequipa | 1238972 | 5·28 | 3787 | 23·51 | 21·90 |
| Ayacucho | 675539 | 1·85 | 642 | 7·3 | 6·85 |
| Cajamarca | 1518210 | 3·84 | 2537 | 12·85 | 12·75 |
| Cusco | 1299526 | 2·05 | 1558 | 9·22 | 9·38 |
| Huancavelica | 488042 | 2·17 | 465 | 7·33 | 7·34 |
| Huanuco | 853222 | 4·11 | 1507 | 13·59 | 14·69 |
| Junin | 1348404 | 2·55 | 1777 | 10·14 | 9·9 |
| Pasco | 298983 | 3·23 | 366 | 22·44 | 24·66 |
| Puno | 1403675 | 2·27 | 2066 | 10·82 | 9·68 |
| Jungle |  |  |  |  |  |
| Amazonas | 431483 | 3·34 | 533 | 9·50 | 11·16 |
| Loreto | 997706 | 1·63 | 546 | 3·92 | 5·31 |
| Madre de Dios | 124889 | 2·04 | 107 | 7·51 | 11·21 |
| San Martin | 805106 | 2·45 | 810 | 7·64 | 9·44 |
| Ucayali | 476706 | 2·90 | 659 | 10·21 | 14·47 |

^a^ ASMR: age-standardized mortality rate. CD: Coronary disease.
